# Structural basis for recognition of the central conserved region of RSV G by neutralizing human antibodies

**DOI:** 10.1371/journal.ppat.1006935

**Published:** 2018-03-06

**Authors:** Harrison G. Jones, Tina Ritschel, Gabriel Pascual, Just P. J. Brakenhoff, Elissa Keogh, Polina Furmanova-Hollenstein, Ellen Lanckacker, Jehangir S. Wadia, Morgan S. A. Gilman, R. Anthony Williamson, Dirk Roymans, Angélique B. van ‘t Wout, Johannes P. Langedijk, Jason S. McLellan

**Affiliations:** 1 Department of Biochemistry and Cell Biology, Geisel School of Medicine at Dartmouth, Hanover, New Hampshire, United States of America; 2 Janssen Vaccines & Prevention, Leiden, The Netherlands; 3 Janssen Prevention Center, Janssen Pharmaceutical Companies of Johnson and Johnson, San Diego, California, United States of America; 4 Janssen Prevention Center, Janssen Vaccines & Prevention B.V., Leiden, The Netherlands; 5 Janssen Infectious Diseases, Janssen Pharmaceutica NV, Beerse, Belgium; 6 Janssen R&D US, San Diego, California, United States of America; 7 Janssen Prevention Center, Janssen Pharmaceutical Companies of Johnson and Johnson, London, United Kingdom; NIH, UNITED STATES

## Abstract

Respiratory syncytial virus (RSV) is a major cause of severe lower respiratory tract infections in infants and the elderly, and yet there remains no effective treatment or vaccine. The surface of the virion is decorated with the fusion glycoprotein (RSV F) and the attachment glycoprotein (RSV G), which binds to CX3CR1 on human airway epithelial cells to mediate viral attachment and subsequent infection. RSV G is a major target of the humoral immune response, and antibodies that target the central conserved region of G have been shown to neutralize both subtypes of RSV and to protect against severe RSV disease in animal models. However, the molecular underpinnings for antibody recognition of this region have remained unknown. Therefore, we isolated two human antibodies directed against the central conserved region of RSV G and demonstrated that they neutralize RSV infection of human bronchial epithelial cell cultures in the absence of complement. Moreover, the antibodies protected cotton rats from severe RSV disease. Both antibodies bound with high affinity to a secreted form of RSV G as well as to a peptide corresponding to the unglycosylated central conserved region. High-resolution crystal structures of each antibody in complex with the G peptide revealed two distinct conformational epitopes that require proper folding of the cystine noose located in the C-terminal part of the central conserved region. Comparison of these structures with the structure of fractalkine (CX3CL1) alone or in complex with a viral homolog of CX3CR1 (US28) suggests that RSV G would bind to CX3CR1 in a mode that is distinct from that of fractalkine. Collectively, these results build on recent studies demonstrating the importance of RSV G in antibody-mediated protection from severe RSV disease, and the structural information presented here should guide the development of new vaccines and antibody-based therapies for RSV.

## Author summary

Respiratory syncytial virus (RSV) is a common cause of bronchiolitis and pneumonia, and is a leading cause of infant deaths globally due to infectious disease. Despite decades of research, there is still no vaccine or widespread treatment for RSV. In this study, we isolated two antibodies that bind to the central conserved region of the viral attachment glycoprotein, RSV G. The antibodies effectively neutralize both subtypes of RSV and protect RSV-challenged animals from severe disease. We also determined high-resolution crystal structures of each antibody in complex with the central conserved region of RSV G to gain a better understanding of how these antibodies bind to RSV G and how they neutralize the virus. Because RSV G is a small folded domain bounded by unstructured mucin-like domains, structural elucidation of the central conserved region provides atomic-level information for the complete folded portion of RSV G. The results presented here will help develop effective antibodies for passive prophylaxis as well as guide efforts to design vaccines that elicit broadly neutralizing antibodies against RSV G.

## Introduction

Human respiratory syncytial virus (RSV) infects nearly all children by the age of two [1]. Although RSV does not typically cause severe disease in healthy adults, it is a major pathogen for infants, the elderly, and immunocompromised individuals, with a propensity to cause severe lower respiratory tract infections and pneumonia [2, 3]. In 2015 alone, RSV is estimated to have caused the deaths of 94,000–149,000 children under the age of five [4]. Although infant deaths in the U.S. due to RSV are rare, it is estimated that each year 2.1 million children under the age of five in the U.S. require medical attention [3]. Currently, there is no vaccine for RSV and the only FDA-approved therapy is passive prophylaxis with the monoclonal antibody palivizumab (Synagis), which binds to the viral fusion (F) glycoprotein [5]. However, the cost and efficacy of palivizumab restricts its use to high-risk infants, leaving a substantial population at risk with no available targeted therapy [6].

RSV is an enveloped, negative-sense single-stranded RNA virus that belongs to the *Pneumoviridae* family. RSV virions have three proteins on the surface: the aforementioned F glycoprotein, the attachment glycoprotein (G), and the small hydrophobic protein (SH) [7]. The F and G glycoproteins play an important role in mediating RSV infection and both are major targets of the humoral immune response [8–10]. RSV, and the related human metapneumovirus (hMPV), do not require the attachment protein for *in vitro* infection, indicating that the fusion protein alone is sufficient to mediate viral attachment and entry [11–14]. In contrast, viruses in the related *Paramyxoviridae* family require the cognate attachment protein for infection, as it relays the signal of receptor binding to the F glycoprotein to initiate membrane fusion at the proper time and place [15–17]. However, despite no absolute requirement for the G glycoprotein among pneumoviruses *in vitro*, infection of humans and animal models appears to be much more dependent upon the presence of G, as ΔG strains are heavily attenuated *in vivo* [12, 13]. Therefore, the G protein could serve as an effective therapeutic target.

RSV infection produces two forms of the G protein: the first is a full-length membrane-bound form that mediates viral attachment; the second is a secreted form (sG) that arises due to translation initiation at an AUG codon in the transmembrane domain [18–20]. Full-length RSV G is a type II membrane protein with 30–40 *O*-linked glycans and 3–5 *N*-linked glycans that contribute ~60% of the glycoprotein’s molecular weight [7, 21–25]. The ectodomain consists of an unglycosylated central conserved region flanked by two hypervariable mucin-like domains that lack structure [7, 26, 27]. The central conserved region contains thirteen amino acids that are strictly conserved across all RSV strains [25], and these residues partially overlap with a cystine noose containing a 1–4, 2–3 disulfide topology, with the residues spanning the third and fourth cysteines (Cys182–Cys186) forming a CX3C motif [26, 28, 29]. The cystine noose is thought to mediate RSV attachment to human airway epithelial cells during natural infection by interaction with CX3CR1 [30–33]. NMR structures of the cystine nooses from human and bovine RSV G revealed that both adopt a similar rigid structure [34, 35]. Immediately downstream of the cystine noose lies the heparin-binding domain, composed of positively charged residues that are involved in attachment to heparan sulfate and other glycosaminoglycans (GAGs) [36, 37]. Although cell-surface GAGs have been shown to facilitate infection of immortalized cell lines, their importance to natural infection is less clear, as human ciliated respiratory epithelial cells do not contain heparan sulfate on their cell surface [38].

The humoral antibody response to RSV is directed equally against the F and G glycoproteins [9]. G-directed human antibodies, such as 3D3 and 3G12, that bind to the central conserved region have been shown to potently neutralize both subtypes of RSV. These two antibodies bind with high affinity to RSV G and protect mice from RSV challenge [39, 40]. Additionally, the central conserved region of G has been shown to induce a persistent and protective antibody response in mice [41–43]. Such studies led to the isolation of a murine antibody, 131-2G, that binds to the central conserved region and blocks the interaction of G with CX3CR1, thereby inhibiting viral attachment [29, 44]. To gain insight into the modes of antibody recognition of the central conserved region of G, we isolated and characterized a panel of human monoclonal antibodies that target G. We identified two antibodies, CB002.5 and CB017.5, that bind with high affinity to sG, potently inhibit infection of human bronchial epithelial cells (HBECs) in the absence of complement, and protect cotton rats from severe RSV disease. Crystal structures of each antibody in complex with a 45-residue G-derived peptide corresponding to the central conserved region revealed two distinct conformational epitopes and demonstrate that the RSV G CX3C motif adopts a different structure than that observed in fractalkine (CX3CL1), the natural ligand of CX3CR1.

## Results

### G-directed antibodies potently neutralize RSV and protect against severe RSV disease

**Fig 1:**
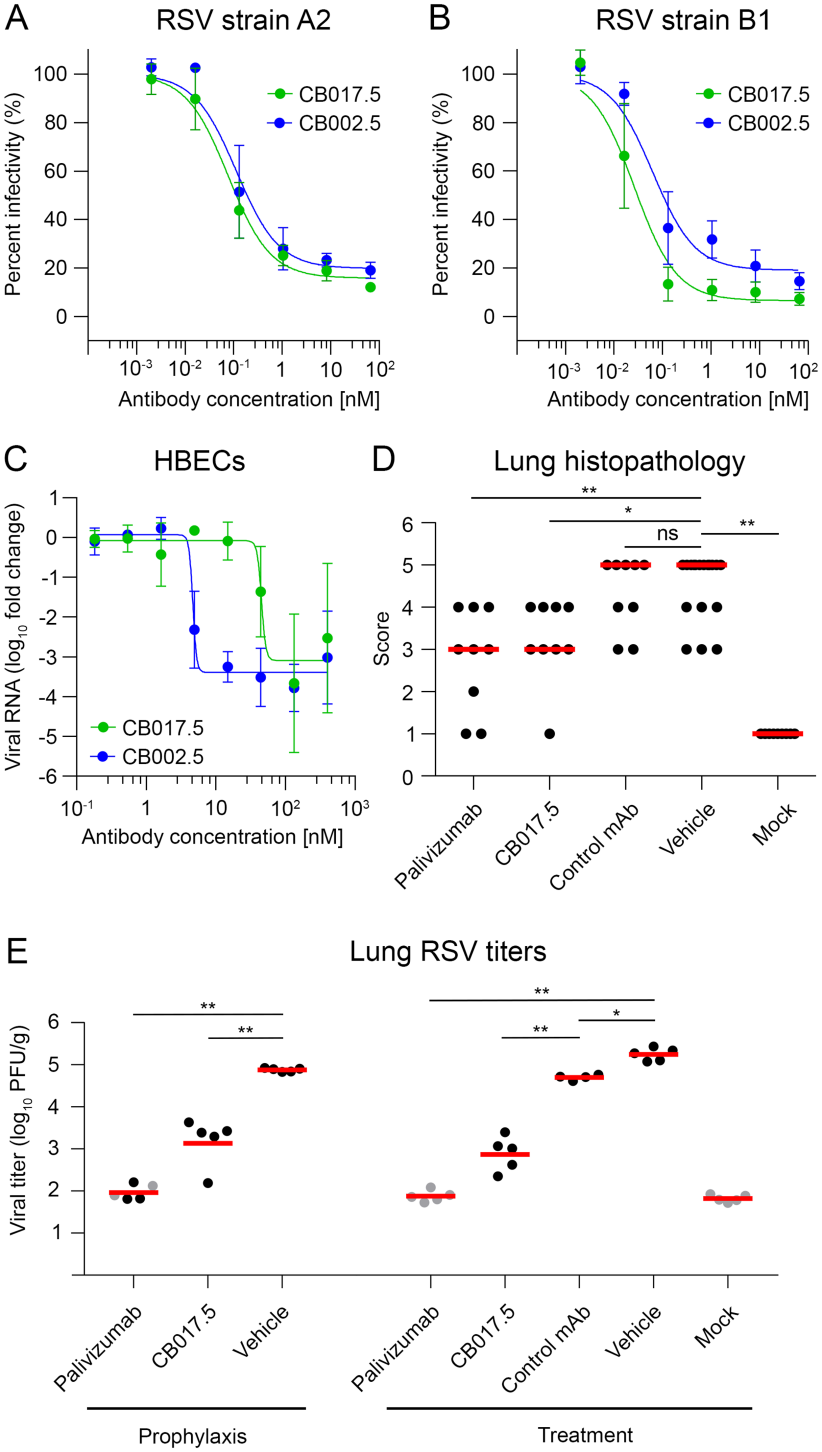
Virus neutralization and cotton rat protection studies. Neutralization curves based upon a plaque-reduction assay performed in Vero cells in the presence of complement using RSV strain A2 (A) or strain B1 (B) when incubated with CB017.5 IgG (green) or CB002.5 IgG (blue). IC_50_ values were determined by the mean concentration required to inhibit 50% of RSV infection. (C) Neutralization curves, in the absence of complement, of an *in vitro* RSV cell culture model using human bronchial epithelial cells (HBECs) cultured at an air-liquid interface, colored as in A and B. (D) Cotton rat lung histopathology scores of each treatment group in the treatment arm of the study were evaluated six days after infection. Slides were scored blindly as described in the methods, with lower scores indicating reduced inflammation and pathology. The red line indicates the median of each group. (E) Infectious RSV titers in the lungs of cotton rats four days post-infection as determined by plaque assay. Different animal groups were injected prophylactically 24 hours prior to infection, or as a treatment one day after infection, as indicated. Animals used to determine viral titers were not included in the histopathological analysis. The red line indicates the mean viral titer. Gray dots indicate viral titers that were at the lower limit of detection. For panels (D) and (E): *p <.05, **p <.01, ns = not significant.

Antibodies CB002.5 and CB017.5 were isolated from healthy, but RSV-experienced, adults. Peptide-mapping data using alanine scanning and binding to short RSV G peptides demonstrated that these two antibodies both bound to the central conserved region of G and have distinct, but overlapping, epitopes (S1 Fig). We performed initial viral neutralization assays in immortalized cells, utilizing both subtypes of RSV. Antibody CB002.5 and antibody CB017.5 were unable to neutralize either subtype of RSV in the absence of complement (S2 Fig). However, in the presence of complement, each antibody potently neutralized both subtypes of RSV. CB002.5 had a 50% inhibitory concentration (IC_50_) of 0.12 nM and 0.06 nM for strains A2 and B1, respectively. Similarly, antibody CB017.5 neutralized RSV with an IC_50_ of 0.08 nM for strain A2 and 0.03 nM for strain B1 (Fig 1A and 1B). We also performed neutralization assays, in the absence of complement, using primary, well-differentiated HBECs cultured at an air-liquid interface (S3 Fig). This system has previously been demonstrated to more accurately represent natural RSV infection and is particularly important for evaluating G-directed antibodies due to the presence of CX3CR1 and lack of heparan sulfate on the cell surface [31, 38, 45]. Antibody CB002.5 demonstrated potent neutralization of RSV in HBECs with an IC_50_ of 4.7 nM, while antibody CB017.5 was approximately 10-fold less effective with an IC_50_ of 45.4 nM (Fig 1C).

To evaluate the clinical potential of G-directed antibodies that bind to the central conserved region, we performed prophylactic and post-challenge treatment experiments using CB017.5 in the RSV cotton rat model (Fig 1D and 1E). Animals in the prophylaxis arm received an antibody injection 24 hours prior to intranasal RSV infection, whereas animals in the treatment arm were injected one day post-challenge. Additional animal groups received palivizumab, vehicle only, an irrelevant antibody, or mock RSV infection as controls. Therapeutic efficacy was evaluated by measuring infectious RSV titers in the lungs as well as histopathology scores to determine the extent of pulmonary inflammation. Antibody CB017.5 reduced infectious viral titers by approximately two orders of magnitude compared to the irrelevant antibody or vehicle-only treatments. CB017.5 also appeared to be equally effective at reducing infectious viral loads both prophylactically and as a post-infection therapeutic, although it appeared less effective than palivizumab in reducing viral load (Fig 1E). However, histopathology scores demonstrated that antibody CB017.5 and palivizumab were comparable in reducing pulmonary inflammation associated with severe RSV disease (Fig 1D). These results demonstrate that antibodies CB017.5 and CB002.5 neutralize RSV infection of primary HBECs in the absence of complement and that CB017.5 effectively protects cotton rats from severe RSV disease.

### Fab CB002.5 and Fab CB017.5 bind with high affinity to RSV G

**Fig 2:**
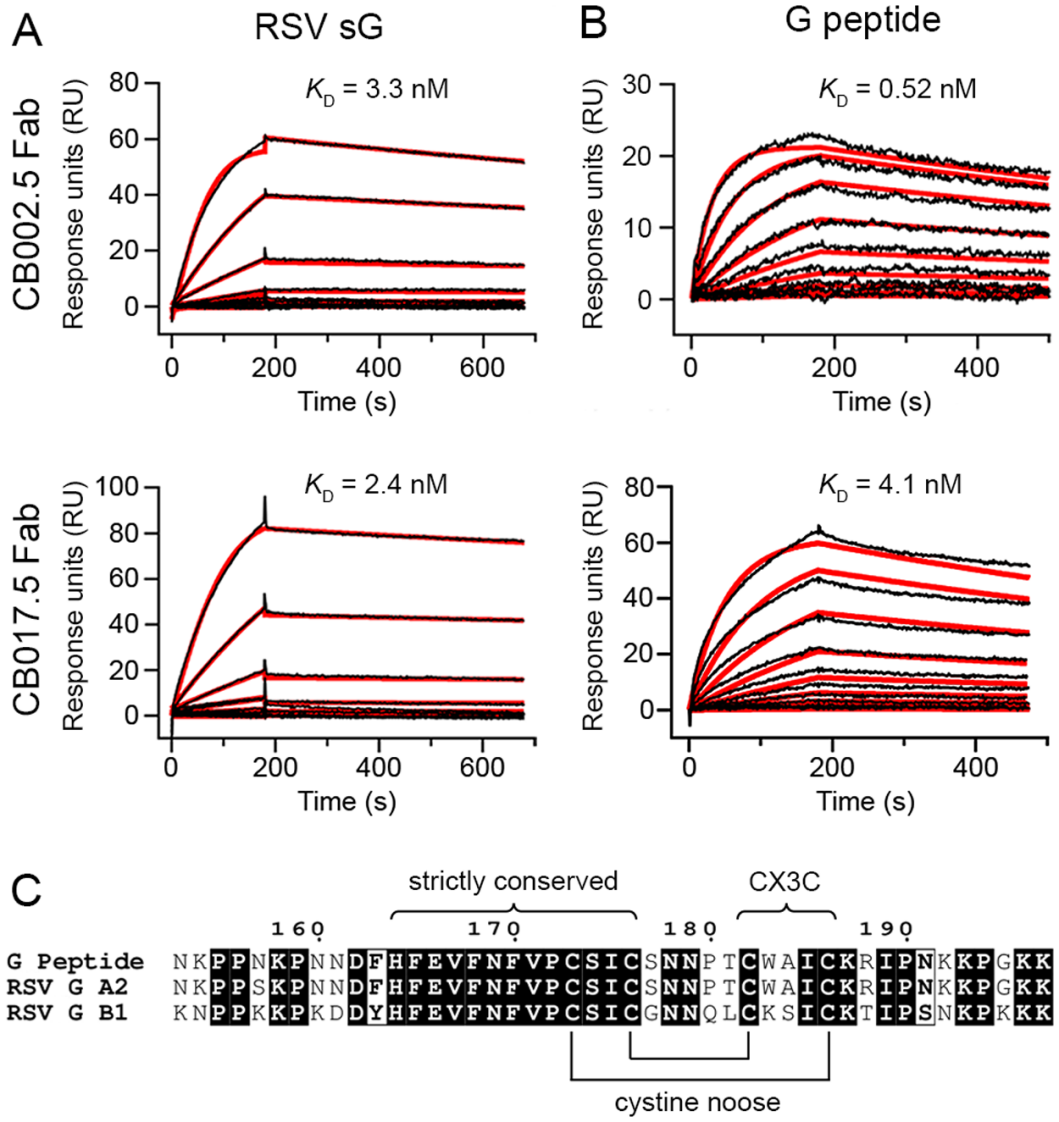
Fabs CB017.5 and CB002.5 bind with high affinity to RSV G and a G peptide. Surface plasmon resonance (SPR) response curves of Fab CB002.5 (top) and Fab CB017.5 (bottom) binding to wild-type RSV sG from strain A2 (A) and a subtype A RSV G peptide encompassing the central conserved region (B). The raw data are plotted in black, and the calculated best fit to a 1:1 binding model is plotted in red. The equilibrium dissociation constant (*K*_D_) for each interaction is displayed above the respective SPR curve. (C) Sequence alignment of the 45-residue G peptide and the corresponding region of RSV G from strains A2 and B1. The strictly conserved residues, the cystine noose, and CX3C motif are labeled.

We performed surface plasmon resonance (SPR) studies to characterize the binding kinetics and affinity of antibodies CB002.5 and CB017.5 for RSV G. We evaluated the interaction of the antigen-binding fragment (Fab) of each antibody with a secreted form of RSV G from strain A2 (sG), as well as with a 45-residue synthetic peptide encompassing the subtype A RSV G central conserved region (G peptide) that contains the strictly conserved residues and cystine noose. Fab CB002.5 and Fab CB017.5 bound to sG with equilibrium dissociation constants (*K*_D_) of 3.3 nM and 2.4 nM, respectively (Fig 2A). These affinities for sG were similar to those determined for binding to the G peptide, which were 0.52 nM for Fab CB002.5 and 4.1 nM for Fab CB017.5 (Fig 2B). That both antibodies bound to sG and the G peptide with comparable affinities suggests that the antibody epitopes lie entirely within the region encompassed by the peptide (Fig 2C).

To investigate the extent to which intact disulfide bonds in the G peptide influence antibody recognition, we performed additional SPR experiments for each Fab with a reduced and alkylated form of the G peptide. Based on an analysis of the binding kinetics, Fab CB002.5 and Fab CB017.5 bound to the reduced and alkylated form of the G peptide with a *K*_D_ of 18,000 nM and 27 nM, respectively (S4 Fig). This represents an approximate 35,000-fold reduction in the affinity of Fab CB002.5 and a 7-fold reduction in Fab CB017.5 affinity, indicating that both Fabs preferentially bind to the G peptide with intact disulfides, with CB002.5 being strongly dependent upon disulfide-bond formation within the cystine noose.

### Crystal structure of Fab CB002.5 in complex with the RSV G peptide

**Fig 3:**
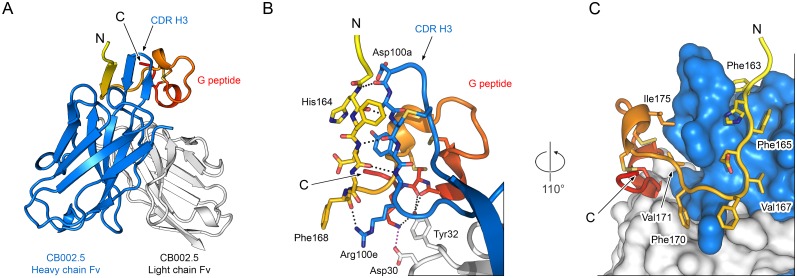
Crystal structure of Fab CB002.5 bound to an RSV G peptide. (A) Ribbon diagram of the Fab CB002.5 variable domain (Fv) in complex with the subtype A RSV G peptide. (B) Ribbon-and-stick representation of the interaction between the CDR H3 of Fab CB002.5 and the G peptide. Black dotted lines indicate hydrogen bonds and the red dotted line indicates a salt bridge. (C) Ribbon-and-stick representation of the G peptide and a molecular surface representation of Fab CB002.5, rotated 110° from the view in (B). In panels (A–C) the heavy chain is colored blue, the light chain is colored white, and the G peptide is colored on a spectrum from yellow to red, N- to C-terminus, respectively. For stick models, oxygen atoms are colored red, nitrogen are blue, and sulfur are yellow.

To characterize the interaction of antibody CB002.5 with the central conserved region of RSV G, we determined the crystal structure of Fab CB002.5 in complex with the G peptide to 2.1 Å resolution (Fig 3, Table 1). The complex crystallized in space group *C*2 and the asymmetric unit contained four copies of the complex, with the only notable discrepancy between the complexes being the elbow angle between the variable and constant domains of the Fab. Overall, the alignment of the variable domains and G peptides of all four complexes resulted in a root-mean-square deviation ranging from 0.13 Å to 0.31 Å. Although the G peptide is 45 residues in length (Asn153–Lys197), the electron density allowed for accurate placement of only 30 residues (Asn160–Ile189), indicating that the N- and C-termini of the G peptide exhibit structural flexibility and that the epitope of Fab CB002.5 is likely confined within these 30 residues. Fab CB002.5 buries 1,067 Å^2^ of surface area on the G peptide, with the majority of the interface formed between the CB002.5 heavy chain and the strictly conserved residues of the RSV G central region (Fig 3A). The heavy chain buries 823 Å^2^ (77%) of the total buried surface area (BSA).

**Table 1:** Crystallographic data collection and refinement statistics.

	Fab CB002.5–G peptide	Fab CB017.5–G peptide
**PDB ID** **Data collection**	6BLI	6BLH
Space group	*C*2	*C*222_1_
Wavelength (Å)	1.033	0.979
Cell dimensions		
*a*, *b*, *c* (Å)	113.8, 85.2, 214.3	85.1, 189.6, 74.1
α, β, γ (°)	90, 95, 90	90, 90, 90
Resolution (Å)	43.1–2.1 (2.16–2.12)*	41.9–2.0 (2.05–2.00)
*R*_merge_	0.170 (0.573)	0.174 (0.487)
*I* / σ*I*	4.0 (1.6)	5.3 (1.8)
CC_1/2_	0.972 (0.596)	0.988 (0.901)
Completeness (%)	99.2 (99.5)	98.5 (98.7)
Redundancy	2.9 (2.8)	5.8 (6.1)
Total reflections	329,996 (15,747)	232,594 (18,016)
Unique reflections	114,818 (5,719)	40,222 (2,939)
**Refinement**		
Resolution (Å)	43.1–2.1 (2.20–2.12)	41.9–2.0 (2.07–2.00)
Unique reflections	114,768 (11,440)	40,202 (3,983)
*R*_work_ / *R*_free_ (%)	16.9/21.8	18.7/22.6
No. atoms	16,015	4,093
Protein	14,178	3,530
Water	1,837	562
EDO	-	4
*B*-factors (Å^2^)		
Protein	23.7	29.4
Water	32.6	37.0
EDO	-	32.4
R.m.s. deviations		
Bond lengths (Å)	0.008	0.002
Bond angles (°)	0.97	0.52
Ramachandran (%)		
Favored	96.8	97.2
Allowed	3.2	2.8
Outliers	0	0

Data were collected from one crystal.

*Values in parentheses are for highest-resolution shell.

One of the prominent interactions observed in the crystal structure involves the third complementarity-determining region of the heavy chain (CDR H3). The CDR H3 of Fab CB002.5 forms a disulfide-stabilized β-hairpin, which in turn makes main-chain hydrogen bonds with the strictly conserved G residues 164-HFEVF-168 to form a parallel β-strand interaction (Fig 3B). In addition, conserved residues of RSV G from the same region, including Phe163, Phe165, Val167, and Phe170, have hydrophobic side chains that fill pockets on the surface of Fab CB002.5 (Fig 3C). Therefore, the stretch of strictly conserved residues of RSV G contribute to the binding interaction with antibody CB002.5 through both main-chain hydrogen bonds and side-chain van der Waals interactions.

The cystine noose appears to play a dual role in Fab CB002.5 binding. The cystine noose lies at the interface of the heavy and light chains of Fab CB002.5, facilitating the interaction of hydrophobic side chains of the G peptide, such as Val171 and Ile175, with the CDR H3 (Fig 3C). In addition, the cystine noose folds the protein back upon itself to form a hairpin, bringing the residues immediately C-terminal to the cystine noose into close proximity with the N-terminal residues and into contact with the Fab, forming a conformation-dependent epitope. Specifically, this brings residues Ile185, Cys186, and Lys187 of RSV G, which are conserved across both RSV subtypes, back to form three hydrogen bonds with Tyr32 and a salt bridge with Asp30 of the CB002.5 light chain, burying an additional ~85 Å^2^ of surface area (Fig 3B). Consistent with SPR experiments, disulfide-bond formation and the cystine noose are thus required for proper recognition of the conformation-dependent epitope by antibody CB002.5.

### Crystal structure of Fab CB017.5 in complex with the RSV G peptide

**Fig 4:**
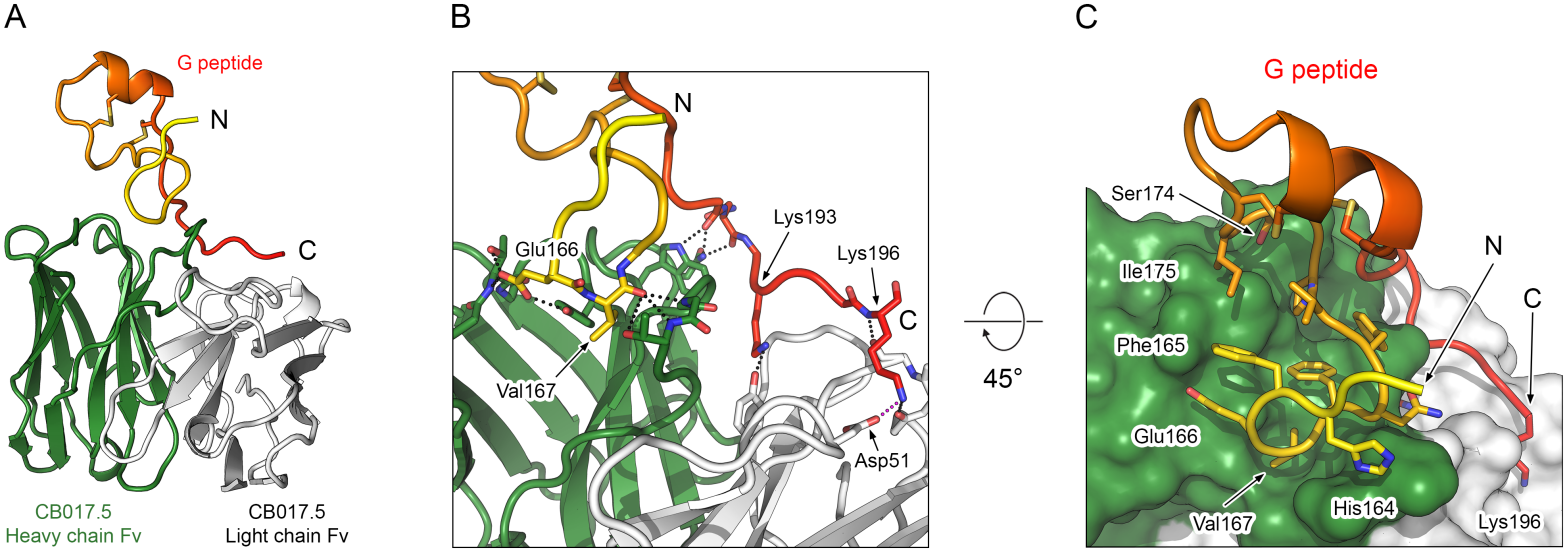
Crystal structure of Fab CB017.5 bound to an RSV G peptide. (A) Ribbon diagram of the Fab CB017.5 variable domain (Fv) in complex with the subtype A RSV G peptide. (B) Ribbon-and-stick representation of Fab CB017.5 in complex with the G peptide. Black dotted lines indicate hydrogen bonds and the red dotted line indicates a salt bridge. (C) Ribbon-and-stick representation of the G peptide and a molecular surface representation of Fab CB017.5, rotated 45° from the view in (B). In panels (A–C) the heavy chain is colored green, the light chain is colored white, and the G peptide is colored on a spectrum from yellow to red, N- to C-terminus, respectively. For stick models, oxygen atoms are colored red, nitrogen are blue, and sulfur are yellow.

To investigate the binding mode of antibody CB017.5 with the central conserved region of G, we determined the crystal structure of Fab CB017.5 in complex with the G peptide to 2.0 Å resolution (Fig 4, Table 1). The complex crystallized in space group *C*222_1_ and contained one complex in the asymmetric unit. Ordered electron density was observed for 35 of the 45 G peptide residues (Asp162–Lys196), including seven C-terminal residues not observed in the Fab CB002.5 complex structure. However, the tip of the cystine noose in this structure showed weak electron density for residues Asn178, Asn179, and Pro180, indicating that it is structurally flexible in this complex. Fab CB017.5 buries a total surface area of 1,081 Å^2^ on the G peptide, primarily mediated through the heavy chain, which buries 720 Å^2^ (67%) (Fig 4A and 4B). Interestingly, Fab CB017.5 has little interaction with the cystine noose itself, but rather establishes the vast majority of contacts with conserved residues N- or C-terminal to the cystine noose, in agreement with peptide binding studies (S1 Fig). The only residues within the cystine noose to contact Fab CB017.5 are Ser174 and Ile175, with their side chains abutting the surface of the heavy chain, but making no additional intermolecular interactions (Fig 4C).

The strictly conserved residues of RSV G from His164–Cys176 account for 443 Å^2^ of the buried surface area and make six hydrogen bonds with the heavy chain of Fab CB017.5: three with the side chain of Glu166 and three with the main-chain carbonyl group of Val167 of the G peptide (Fig 4B). Additionally, Fab CB017.5 has a substantial interface with the residues C-terminal to the cystine noose, burying 638 Å^2^ of surface area extending from Arg188 to Lys196 and forming hydrogen bonds with Pro190, Asn191, Lys192, Lys193, and Lys196 of the G peptide. This indicates that the cystine noose contributes to the proper conformation of the epitope by bringing the conserved N- and C-terminal flanking residues into close proximity. However, the modest reduction in affinity when the disulfide bonds within the cystine noose are disrupted (S4B Fig) is consistent with the limited interactions between Fab CB017.5 and the cystine noose itself.

### Structural comparison of the RSV G cystine noose and antibody binding modes

**Fig 5:**
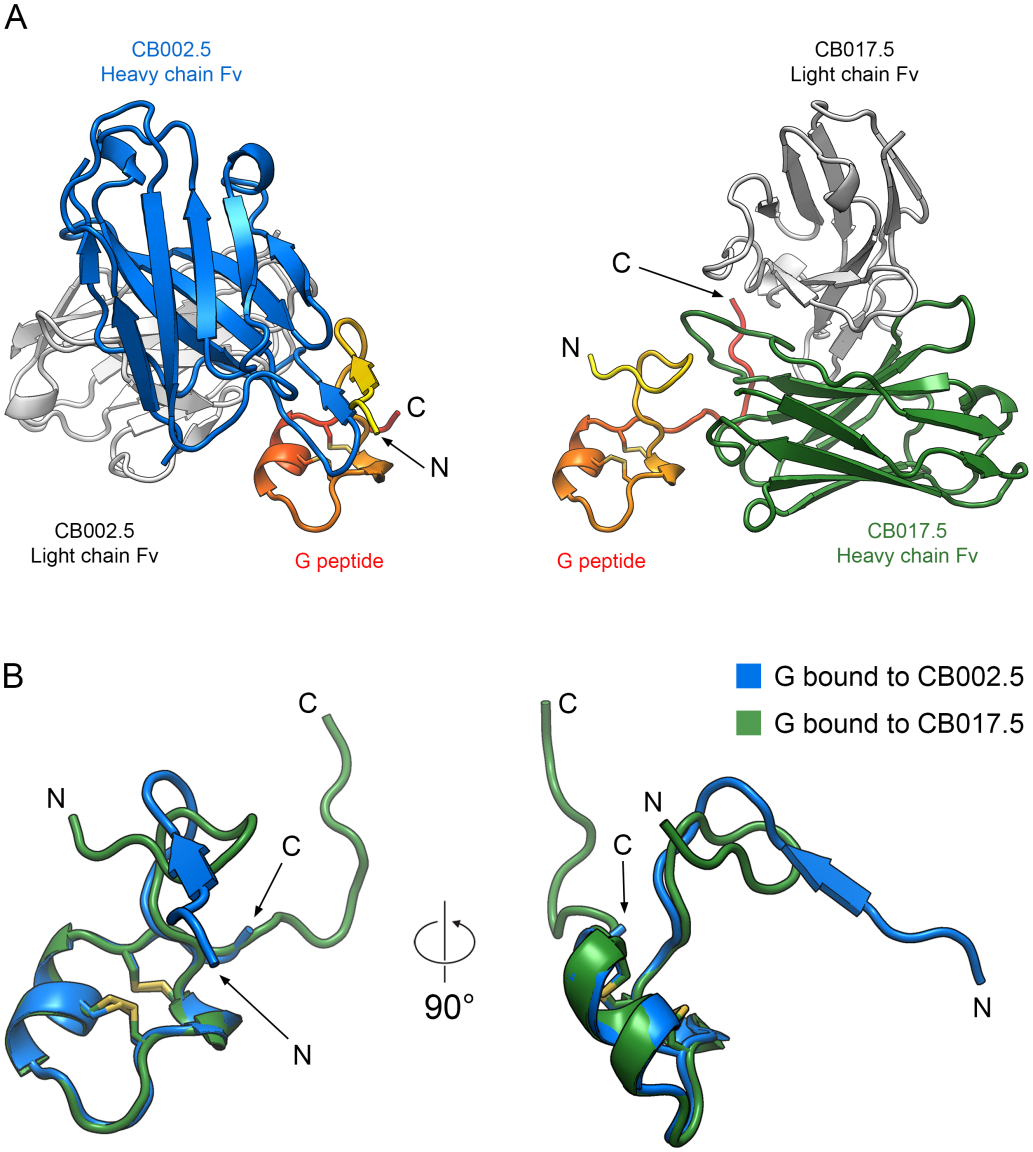
Structural comparison of the Fab–G peptide complexes. (A) Ribbon representation of the Fab CB002.5–G peptide (left) and Fab CB017.5–G peptide (right) complexes, generated by superimposing the structurally conserved cystine noose. Each complex is shown separately for clarity and colored as in Figs 3 and 4. (B) Ribbon representation of the G peptide, shown in the conformation adopted when bound to Fab CB002.5 (blue) or Fab CB017.5 (green). The figure was generated by superimposing the cystine nooses of the G peptides (Cys173–Cys186). The two disulfide bonds are shown as sticks, with sulfur atoms colored yellow.

Despite antibodies CB002.5 and CB017.5 both targeting the central conserved region of RSV G, there are several key differences in antibody binding, including the angle of approach by each antibody and the structural conformations adopted by the central region. Overlaying the cystine noose from each crystal structure highlights the large difference in the angle of approach, with the two antibodies approaching G from opposite directions (Fig 5A). In addition, overlaying the G peptides from both crystal structures demonstrates that the cystine noose is structurally rigid, in agreement with previous NMR structures of this region in solution (Fig 5B) [34, 35]. However, structural comparison of the strictly conserved residues N-terminal to the cystine noose highlights the flexibility of G. Despite strict sequence conservation across all RSV strains, the N-terminal portion of the central conserved region is flexible and adopts distinct conformations in the two different complexes (Fig 5B). Similarly, the structure of the G peptide bound to Fab CB002.5 shows no electron density beyond Ile189, suggesting that the C-terminal region beyond the cystine noose is also inherently flexible. Collectively, these structures suggest that the cystine noose is a rigid structural element with some flexibility in the tip of the noose, and that the regions flanking the noose are conformationally heterogeneous.

### RSV G and fractalkine CX3C motifs are structurally dissimilar

**Fig 6:**
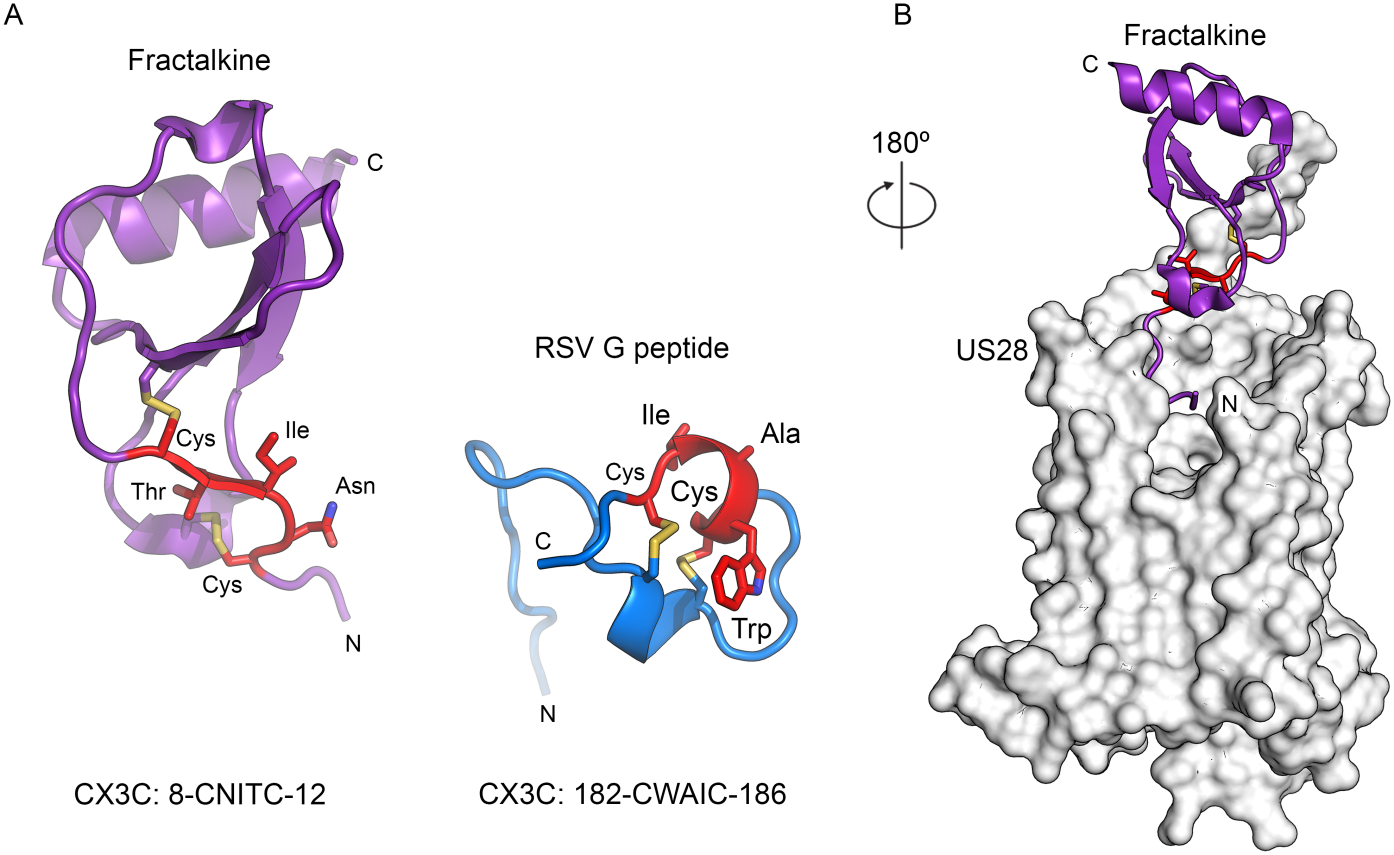
Structural comparison of RSV G and fractalkine CX3C motifs. (A) Ribbon diagram of fractalkine (left, purple, PDB ID: 1F2L) and the RSV G peptide, shown in the conformation adopted when bound to Fab CB002.5 (right, blue). The CX3C motif is displayed as red sticks and the sequence is shown below each structure. (B) Ribbon diagram of fractalkine bound to US28, a viral-homolog of human CX3CR1 (shown as a white molecular surface; PDB ID: 4XT1), rotated 180° from the view of fractalkine in (A). For stick models in panels (A) and (B), oxygen atoms are colored red, nitrogen are blue, and sulfur are yellow.

The structural basis for the interaction of RSV G with CX3CR1 remains poorly understood. Several groups have noted that the RSV G cystine noose contains a CX3C motif, suggesting that the cystine noose binds to CX3CR1 in a manner similar to that of fractalkine, the natural ligand of CX3CR1 [29, 46]. However, comparison of the antibody-bound structures of the RSV G central conserved region to that of previously determined structures of fractalkine, both alone and in complex with US28 (a viral homolog of CX3CR1), indicates that the CX3C motif of G does not structurally mimic the CX3C motif of fractalkine (Fig 6A and 6B) [47, 48]. The CX3C motif of fractalkine has a different disulfide-bond connectivity (1–3, 2–4) and exists in an extended conformation, whereas the CX3C motif of RSV G is α-helical. This structural dissimilarity suggests that the presence of a CX3C motif in RSV G and fractalkine may be coincidental, and that RSV G likely binds CX3CR1 in a manner that is substantially different than that of fractalkine.

## Discussion

RSV G plays an important role in RSV entry by mediating attachment of the virus to human airway epithelial cells in a process that involves CX3CR1 [18, 30–33]. Here we describe the isolation and characterization of two human antibodies, CB002.5 and CB017.5, from healthy adults who have previously experienced natural RSV infection. These two antibodies bind to the central conserved region of G and neutralize RSV *in vitro* as well as protect cotton rats from severe RSV disease. Whereas these antibodies potently neutralize RSV infection of HBECs in the absence of complement, they require the presence of complement to neutralize RSV infection of immortalized cells. These findings are consistent with results from other researchers and indicate that different regions of RSV G contact different receptors to mediate attachment to HBECs and immortalized cells [31]. In immortalized cells, the heparin-binding domain of G mediates viral attachment via interactions with heparan sulfate and GAGs [36, 37]. In contrast, heparan sulfate is not detectable on the surface of human airway epithelial cells and infection is facilitated by the interaction of RSV G with CX3CR1, which has been shown to depend upon the cystine noose [31, 32, 38]. CB002.5 is 10-fold more potent than CB017.5 in neutralizing RSV infection of HBECs, indicating that it more effectively inhibits the interaction between G and CX3CR1. This difference in potency is likely due to the larger interface of CB002.5 with the cystine noose of G, whereas CB017.5 has limited interactions with the cystine noose itself.

These antibodies appear to be similar to previously described antibodies, such as L9, 131-2G and 3D3, which also bind to the central conserved region of RSV G and potently neutralize both subtypes of RSV [29, 31, 40, 49]. In addition to neutralizing RSV by preventing viral attachment, these antibodies have been shown to drastically reduce inflammatory cytokine production and leukocyte migration associated with sG–CX3CR1 interactions, which in turn reduces lung inflammation and pathology associated with severe RSV disease [29, 39, 42, 50, 51]. This dual functionality of antibodies that target the central region of RSV G makes them particularly promising candidates for passive prophylaxis.

To better define the structural basis and mechanism of RSV-neutralization by this class of antibodies, we determined the crystal structures of the RSV G central conserved region in complex with antibody CB002.5 and CB017.5. Our results indicate that the cystine noose is structurally conserved, but the remainder of the central conserved region of G is flexible, as demonstrated by the conformational heterogeneity observed in our crystal structures. These results agree with previous NMR structures of peptides encompassing the central conserved regions of human and even bovine RSV G [34, 35]. Our findings are also consistent with previous studies that suggest RSV G is composed mostly of intrinsically disordered, highly glycosylated mucin-like domains [25–27, 52]. Our structures show that the thirteen strictly conserved residues of RSV G in the N-terminal half of the central conserved region do not adopt a conserved structure, but rather are flexible and adopt different conformations depending upon the bound antibody. The role of these conserved residues in receptor binding and RSV attachment remains unknown. Future structural studies of the central conserved region bound to additional neutralizing antibodies or to CX3CR1 will help better define the role of the strictly conserved residues and cystine noose in RSV attachment.

RSV continues to cause a heavy disease burden globally despite intense efforts to produce an effective vaccine or therapy. Identification of the structurally conserved elements of RSV G, as well as the biophysical and structural characterization of G-directed neutralizing antibodies, will help inform future therapeutic development. In particular, our results will guide structure-based vaccine-design efforts to generate a protein scaffold which properly presents the central conserved region of RSV G without the hypervariable and heavily glycosylated mucin-like domains. It will also facilitate understanding of G-mediated viral attachment as well as the underlying mechanisms of neutralizing antibodies that target G, which will help evaluate future vaccine candidates and G-based therapeutics. Additionally, RSV F is a major target of potently neutralizing antibodies, with many F-based therapies and vaccines currently in clinical trials [53]. Cocktail therapies of multiple antibodies have shown to be effective for a variety of viruses including Ebola [54] and HIV-1 [55], and a combined approach utilizing antibodies against both F and G could prove useful for combating RSV.

## Methods

### Ethics statement

Animal experiments were performed under veterinary supervision in accordance with National Institutes of Health guidelines and Sigmovir Institutional Animal Care Utilization Committee’s approved animal study protocol #2. Human blood was obtained through the San Diego Blood Bank and peripheral blood mononuclear cells (PBMCs) were isolated using standard methods. The use of samples from human volunteers followed protocols approved by the San Diego Blood Bank Review Board and informed consent was obtained from the donors prior to the blood donation. All samples were anonymized. HBECs (MucilAir) were purchased from Epithelix Sarl (Geneva, Switzerland), with the inserts containing epithelium of 14 donors. All samples were anonymized.

### Production and labeling of RSV G antigens

C-terminally Myc-tagged and 6xHis-tagged RSV G A/Long (RSV Ga) and B1 (RSV Gb) proteins lacking the transmembrane domain (RSV Ga, amino acids 65–288; and RSV Gb, amino acids 65–299) and with the human V kappa I signal peptide to promote secretion were cloned into pcDNA3.1 (Thermo Fisher). Constructs were transfected into HEK293 cells (ATCC CRL-1573), and supernatants were harvested 72 hours post-transfection and dialyzed overnight against 20 mM Tris-HCl pH 8.0 and 300 mM NaCl. Proteins were purified by Ni-NTA chromatography according to the manufacturer’s recommendations (Qiagen Corp.). Protein was dialyzed against phosphate-buffered saline (PBS) at 4°C overnight. Dialyzed proteins were then concentrated, quantified, and fluorescently labelled with Alexa Fluor 647 (AF 647, Thermo Fisher) or Alexa Fluor 488 (AF 488, Thermo Fisher) for RSV Ga and Gb, respectively, for recovery of antigen-specific memory B-cells.

### Isolation of antibodies CB002.5 and CB017.5

CD22^+^ B cells were magnetically enriched (Miltenyl Biotec) and stained with fluorescently labeled antibodies to B-cell surface markers (IgG-FITC, CD19-PerCP-Cy5.5 and CD27-PE-Cy7; BD Biosciences) as well as the fluorescently tagged recombinant RSV Ga and Gb proteins. Doublets and dead cells were excluded. CD19^+^, IgG^+^, CD27^+^, Ga/Gb double-positive cells were collected by single-cell fluorescence-activated cell sorting (FACS) into PCR plates containing RT-PCR reaction buffer and RNaseOUT (Thermo Fisher). cDNA was generated using Superscript III First Strand Synthesis kit (Invitrogen). 2.5 μL of the cDNA was used as a template to amplify the heavy chain as well as the kappa and lambda light chain variable regions using a two-step PCR approach with Platinum Pfx polymerase (Thermo Fisher). A pool of leader specific 5′ primers and a single 3′ primer designed to the C_H_1 region of the heavy chain, C_κ_ region of the kappa light chain, and C_λ_ region of the lambda light chain, were used in the first step of the PCR. Framework-specific 5′ primers and a pool of reverse primers specifically designed to the junction regions of the heavy and light chains were used for the second PCR reaction. Variable fragments were subsequently linked into a single cassette via overlap extension PCR and subcloned into a bacterial Fab expression vector. Bacterial colonies were picked and grown overnight to express Fabs. Bacterial pellets were lysed, spun down, and supernatants were tested for binding to RSV Ga or RSV Gb by enzyme-linked immunosorbent assay (ELISA). Heavy and light chain variable regions of confirmed binders were then sequenced and cloned into mammalian expression vectors, followed by expression and purification by Protein A chromatography as full-length IgG1s. A total of 152 RSV Ga and Gb-specific Fabs were converted into IgGs, expressed, and purified. Each IgG was assessed by SDS-PAGE and size-exclusion chromatography, and titrated against RSV Ga and Gb antigens by ELISA. Finally, each IgG was quantified by three independent measurements and averaged (UV spectrophotometry, BCA assay, and via Octet). Of the 152 monoclonal antibodies (mAbs) tested, 34 had lower IC_50_ values compared to the benchmark (3D3 IgG, Trellis Bioscience) against both RSV strains A2 and B1.

### Pepscan analysis

The binding of mAb to peptides was assessed in a Pepscan-based ELISA. Each mAb was titrated to ensure that optimal binding was achieved and nonspecific binding was avoided. Each of the wells in the card contained covalently linked peptides that were incubated overnight at 4°C with mAb at a concentration between 1 and 10 ng/mL in PBS containing 5% (*v/v*) horse serum, 5% (*w/v*) ovalbumin (OVA), and 1% (*v/v*) Tween 80, or in an alternative blocking buffer of PBS containing 4% (*v/v*) horse serum and 1% (*v/v*) Tween 80. After washing, the plates were incubated with a horseradish peroxidase (HRP)-linked rabbit anti-human IgG antibody (DakoCytomation, Glostrup, Denmark) for 1 hr at 25°C. After further washing, peroxidase activity was assessed using ABTS substrate, and color development was quantified using a CCD camera and an image-processing system.

### Production of RSV G peptide

The G peptide was synthesized by standard Fmoc solid-phase peptide synthesis using Rink resin or in protected form on Sasrin resin (Bachem) on a Symphony or Prelude-synthesizer (Protein Technologies), respectively. N-terminal biotin coupling was done as the last step in synthesis. The crude peptide was purified by reverse-phase high-performance liquid chromatography (HPLC). The correct molecular mass of the peptide was confirmed by electro-spray ionization mass spectrometry on an Aquity SQD mass spectrometer (Waters). Peptide 4-acetamidothiophenol thioesters were prepared from the protected peptide by addition of two equivalents of 4-acetamidothiophenol and activation with PyBop (benzotriazol-1-yl-oxytripyrrolidinophosphonium hexafluorophosphate) in dichloromethane with 1% *N*,*N*-diisopropylethylamine. After conversion into the desired thio-ester, the peptide was deprotected in trifluoroacetic acid (TFA):water:triisopropylsilane in a 95:2.5:2.5 ratio. Peptide quality was analyzed by HPLC/MS. Analyses were performed on an Aquity HPLC/MS system using a BEH C18 column (Waters) with a linear gradient 5–55% B in A in 2 min, where solvent A is 0.05% TFA in water and solvent B was 0.05% TFA in acetonitrile. All UV chromatograms were recorded at 215 nm. Oxidative folding was performed to properly form the disulfide bonds of the cystine noose. Standard folding was performed by dissolving 2 mg/mL of reduced peptide in folding buffer (55 mM Tris-HCl, pH 8.0; 150 mM NaCl), followed by rapid dilution into 11 volumes of the same buffer, supplemented with reduced glutathione (GSH) to reach a final concentration of 2 mM, and oxidized glutathione (GSSG) to reach a 0.5 mM final concentration.

### Production of reduced and alkylated G peptide for SPR

RSV G peptide produced according to the above protocol was used to generate a reduced and alkylated form of the G peptide. The G peptide was reduced by incubation with a final concentration of 10 mM TCEP (Tris(2-carboxyethyl)phosphine hydrochloride, Sigma) for 1 hr at 54°C, followed by alkylation with the addition of iodoacetamide (Sigma) to a final concentration of 18 mM and incubation for 1 hr in the dark at room temperature. The reduced and alkylated form of the G peptide was dialyzed overnight in PBS, followed by primary-amine coupling to a CM5 chip and evaluation by SPR.

### Production of RSV sG protein for SPR

A plasmid encoding the secreted form of RSV G from strain A2 (sG) was codon-optimized and purchased from GenScript, cloned into an expression plasmid with a C-terminal 8xHis tag and *Strep*-TagII, and subsequently transfected into FreeStyle 293-F cells (Invitrogen). After six days, cell supernatants were purified over *Strep*-Tactin resin (IBA), and subsequently purified by size-exclusion chromatography (SEC) using a 16/600 Superdex 200 column (GE Healthcare Biosciences) in a buffer of 2 mM Tris-HCl pH 8.0, 200 mM NaCl, and 0.02% NaN_3_. Peak fractions were pooled, concentrated, and snap frozen in liquid nitrogen prior to long-term storage at -80°C.

### Production of CB002.5 and CB017.5 IgGs and Fabs

Plasmids encoding the heavy and light chains of CB002.5 IgG or CB017.5 IgG were transfected into FreeStyle 293-F cells (Invitrogen) or PER.C6 cells. IgGs were purified from the cell supernatants using a Protein A Agarose column (ThermoFisher), which was eluted using 0.1 M glycine pH 3.0 into a buffered solution containing 1/10 (*v/v*) 1 M Tris-HCl pH 8.0. Fabs were generated by papain digestion of the IgG using a 1:100 (*w/w*) ratio of papain:IgG, incubated at 37°C for 48 hr. The Fc and undigested IgG was removed by flowing the digested solution over a Protein A Agarose column. The flow-through was collected and the Fabs purified by SEC using a 16/600 Superdex 75 column (GE Healthcare Biosciences) in PBS.

### Production of Fab CB002.5–G peptide and Fab CB017.5–G peptide complexes

Lyophilized 45-residue G peptide encompassing the sequence of RSV G from subtype A was resuspended in PBS and mixed with Fab CB002.5 or Fab CB017.5 at a 2:1 molar excess. The complex was incubated for 30 minutes at room temperature before final purification of the Fab–G peptide complex by SEC using a 16/600 Superdex 75 column (GE Healthcare Biosciences) in crystallization buffer (see “Crystallization and data collection” section below). Peak fractions were pooled, concentrated, snap frozen in liquid nitrogen, and stored at -80°C prior to crystallization.

### Viral neutralization assay in A549 cells in the absence of complement

Viral neutralization by recombinant antibodies in the absence of complement was determined based upon a microneutralization assay using Firefly Luciferase (FFL)-labeled RSV CL57 grown in A549 cells. Recombinant RSV-directed antibodies were prediluted to 100 μg/mL in Dulbecco's Modified Eagle Medium (DMEM, Gibco) supplemented with 10% fetal bovine serum (FBS, Gibco) and 1% penicillin-streptomycin (Gibco). In half-area white cell culture microplates (Greiner-Bio, Cat #675083) a 9-step dilution bracket (3-fold dilutions each) of recombinant IgG was mixed 1:1 with 25,000 plaque-forming units (PFU) of RSV CL57 FFL and incubated for 1 hr at room temperature. Subsequently, 5 x 10^3^ A549 cells were added to each well, resulting in a multiplicity of infection (MOI) of 5 and a final starting concentration of 25 μg/mL for the recombinant antibodies. Plates were incubated for 20 hr at 37°C and 10% CO_2_. After incubation, the supernatant was discarded and 25 μL of PBS was added to each well, followed by 25 μL of Neolite substrate (Perkin Elmer, Cat #6016711). Each antibody concentration was performed in duplicate and the luminescence signal was determined using the Synergy Neo plate reader. Prism was used to plot the luminescence signal (y-axis) and the antibody concentration in ng/mL (x-axis).

### Viral neutralization assay in Vero cells

Vero cells (ATCC CCL-81) were seeded in 24-well plates (7.5 x 10^4^ cells/well) and allowed to settle overnight. RSV strains A2 (ATCC VR-1540) or B1 (ATCC VR-1580) were diluted to 75 PFU/well in 150 μL total volume per well in Eagle’s Minimum Essential Medium (EMEM, ATCC) containing 10% baby rabbit complement (AbD Serotec). A 7-step dilution bracket (8-fold dilutions each) of recombinant RSV G-directed IgG, started at 20 ng/μL, was combined 1:1 with the diluted RSV mixtures and incubated for 2 hr at 37°C. Each plate in the neutralization assay included a no-antibody control, performed in duplicate. The RSV–IgG mixture was added to Vero cells and incubated for 1 hr with rocking every 15 minutes. Following virus incubation, 1 mL of CMC media (1% methyl cellulose in DMEM containing 2% FBS, 1% penicillin-streptomycin, and 1% L-glutamine) was overlaid on the cells, and the plates incubated at 37°C. Depending on the RSV strain, cells were fixed on day 4 (strain A2) or day 5 (strain B1) with 10% formalin for 1 hr at room temperature. Wells were washed 10 times with distilled water and blocked with 5% nonfat dry milk in PBS for 1 hr. Fixed cells were probed with HRP-conjugated goat anti-RSV antibody (AbCam, ab20686) for 2 hr, and developed with TrueBlue peroxidase substrate (KPL, Cat #50-78-02) for 10 minutes. The number of plaques per well were counted and used to calculate the IC_50_ in Prism by plotting percent infectivity (y-axis) and antibody concentration (x-axis) using sigmoid dose response analysis to fit the curve.

### Viral neutralization assay in HBEC cultures

The RSV-neutralizing capacity of each IgG was also tested in HBECs infected by rgRSV224, which was licensed from the National Institutes of Health [56]. Ready-to-use MucilAir HBECs (Epithelix Sarl) were delivered and maintained at an air-liquid interface according to the manufacturer’s instructions for 1 week prior to the start of the experiment. HBEC cultures each contained ~500,000 well-differentiated respiratory epithelial cells consisting of ciliated cells, goblet cells, and basal cells. Each epithelial cell culture was tested by the manufacturer to ensure ciliary beating, proper sealing of the epithelial layer, mucus production, and proper morphology for respiratory epithelium. To prevent fungal infection, ketoconazole (2 μg/mL) was added to the medium. Prior to the start of the experiment, inserts were washed once with PBS (with Ca^2+^ and Mg^2+^) to remove mucus and cell debris. Cells were infected with rgRSV224 at a MOI of 0.1 by adding 50 μL of virus suspension, supplemented with the appropriate antibody concentration, to the apical compartment and incubating for 1 hr at room temperature. Treatment with antibody was repeated at 10 hr post-infection. After 1 hr of incubation, antibody and virus were removed and all inserts were washed 3 times with medium to remove any unbound virus. After the final wash, the apical side of the culture was exposed to air. Negative controls were mock-infected with medium. Positive controls were infected and supplemented by PBS only (vehicle). 96 hr post-infection, apical wash (D-PBS, 200 μL/insert) of the epithelium was used to determine the amount of released viral RNA by quantitative reverse transcriptase polymerase chain reaction (qRT-PCR) as described previously [57].

### Animal protection experiments

Animal experiments were carried out at Sigmovir Biosystems Inc., Rockville, MD, USA. Male cotton rats (*Sigmodon hispidus*), inbred, seronegative for paramyxoviruses, and 6–8 weeks old, were blindly dosed prophylactically or therapeutically with palivizumab (Synagis, Brocacef, Netherlands), CB017.5, an irrelevant control mAb, or vehicle. In the prophylactic arm, mAbs at 5 mg/kg (n = 5/group), or vehicle (20 mM sodium acetate pH 5.5, 75 mM NaCl, 5% sucrose, 0.01% (*w/v*) Tween 80, n = 5), were dosed intramuscularly (IM) in the upper hind leg 24 hr before challenge with 5.4 log_10_ PFUs of RSV A/Long virus (ATCC VR-26; propagated in HEp-2 cells (ATCC CCL-23)) on day 0. On day 4 post challenge, viral titers in the lung were measured for all five animals in each group of the prophylaxis arm. On day 0 and 4, blood was collected to measure serum antibody concentrations. In the therapeutic arm, animals were challenged on day 0 with 6.1 log_10_ PFUs of RSV A/Long virus, followed by an intra-cardiac injection of the mAbs at 50 mg/kg (n = 14/group), irrelevant control mAb at 50 mg/kg (n = 14), or vehicle (n = 23), on day 1 post challenge. One group of cotton rats was mock challenged (n = 14), followed by a single intra-cardiac injection of vehicle on day 1 post-challenge, to obtain baseline values. On day 4, five animals per group were sacrificed to determine viral titers in the lungs. On day 6, the remaining animals of each treatment group (n = 9 for mAb treatments, n = 18 for vehicle) were sacrificed for histopathological analysis of the lungs. Lung tissues were fixed in formalin and embedded in paraffin for histology. Paraffin-embedded tissues were cut into 5 μm sections, deparaffinized and stained with hematoxylin and eosin. Slides were blinded and examined microscopically. Four parameters of pulmonary inflammation were evaluated: peribronchiolitis (inflammatory cell infiltration around the bronchioles), perivasculitis (inflammatory cell infiltration around the small blood vessels), alveolitis (inflammatory cells within the alveolar spaces), and interstitial pneumonitis (inflammatory cell infiltration and thickening of alveolar walls). Slides were scored blindly on a 0–4 severity scale for each parameter, and the resulting four scores per slide were added up to form the total histopathology score. Blood samples were collected on day 2 and 4 (n = 5) or on day 2 and 6 (n = 9 or 18) to measure serum antibody concentrations.

### Surface plasmon resonance

A secreted form of RSV G (sG) from strain A2 containing a C-terminal 8xHis tag was immobilized on a NTA sensor chip to a total of 60–150 response units (RUs) using a Biacore X100 (GE Healthcare Biosciences). For assays involving RSV G peptides, the G peptide was immobilized on a CM5 chip via primary-amine coupling to a total of 25 RUs. For all assays, a buffer-only sample was first injected over the sample and reference flow cells. For the sG assays, response curves for serial 3-fold dilutions of each Fab from 300 nM to 46.5 pM in HBS-P+ were evaluated, with a duplication of the 11.1 nM concentration. For the RSV G peptide and Fab CB002.5, we performed serial 2-fold dilutions of the Fab from 25 nM to 98 pM in HBS-P+, with a duplication of the 12.5 nM concentration. For the RSV G peptide and Fab CB017.5, serial two-fold dilutions of the Fab from 100 nM to 195 pM in HBS-P+ were used, with a duplication of the 25 nM concentration. For SPR experiments using the reduced and alkylated form of the G peptide, serial two-fold dilutions of Fab CB002.5 from 1,000 nM to 1.95 nM, and Fab CB017.5 from 62.5 nM to 1.95 nM, were performed in HBS-P+ with duplication of the 62.5 nM concentration. All data were double-reference subtracted and fit to a 1:1 binding model using the BIAevaluation or Scrubber2 analysis software. Final binding curves were displayed using GraphPad Prism Version 7.03 for Windows.

### Crystallization and data collection

The best-diffracting crystals of the Fab CB002.5–G peptide complex were produced via hanging-drop vapor diffusion. We mixed 1 uL of Fab CB002.5–G peptide complex (15.0 mg/mL in 50 mM NaCl, 2 mM Tris-HCl pH 8.0, 0.02% NaN_3_) with 1 uL of reservoir solution containing 14.9% (*w/v*) PEG 3350 and 0.1 M succinic acid. Large, flat crystal plates formed after several days and were looped from the drop and briefly transferred to a cryoprotectant solution containing 30% (*w/v*) PEG 3350 and 0.1 M succinic acid before being plunge-frozen in liquid nitrogen. Data were collected at the 23-ID-D beamline (Advanced Photon Source, Argonne National Laboratory).

The best-diffracting crystals of the Fab CB017.5–G peptide complex were produced via hanging-drop vapor diffusion. We mixed 1 uL of Fab CB017.5–G peptide complex (7.5 mg/mL in 200 mM NaCl, 2 mM Tris-HCl pH 8.0, 0.02% NaN_3_) with 1 uL of reservoir solution containing 23.7% (*v/v*) isopropanol, 11.9% (*w/v*) PEG 4000, and 0.1 M HEPES pH 7.5. Small and medium plate-like crystals formed after several days and were looped from the drop and briefly transferred to a cryoprotectant solution containing 20% (*w/v*) PEG 4000, 15% (*v/v*) ethylene glycol, and 0.1 M HEPES pH 7.5. Data were collected at the 19-ID-D beamline (Advanced Photon Source, Argonne National Laboratory).

### Structure determination

Software used in this project was curated by SBGrid [58]. Diffraction data were processed using the CCP4 software suite [59]: data were indexed and integrated in iMOSFLM [60] and scaled and merged with AIMLESS [61]. A molecular replacement solution for the 2.1 Å dataset of the Fab CB002.5–G peptide complex was found by PHASER [62] using a chimeric protein model consisting of the heavy chain of PDB ID: 4NPY and the light chain of PDB ID: 3NA9, separated into the Fv and Fc domains as search models. The structure was built manually in Coot [63] and refined using PHENIX [64] to an *R*_work_/*R*_free_ of 16.9%/21.8% (PDB ID: 6BLI, Table 1).

A molecular replacement solution for the 2.0 Å dataset of the Fab CB017.5–G peptide complex was obtained by PHASER using a chimeric protein model consisting of the heavy chain of PDB ID: 8FAB and the light chain of PDB ID: 4HPY, separated into the Fv and Fc domains as search models. The structure was manually built in Coot and refined using PHENIX to an *R*_work_/*R*_free_ of 18.7%/22.6% (PDB ID: 6BLH, Table 1).

### Statistical analysis

Collected HBEC data were log-transformed to reach normal distribution. Statistical analysis was performed by one-way ANOVA, followed by Dunnett’s post-hoc test. In the *in vivo* cotton rat experiments, infectious virus titers were analyzed using a censored regression model to account for values at the lower limit of detection and groups were compared using a log-likelihood ratio test. First, model validity was assessed, separately for the prophylactic and therapeutic arm, by a comparison between the palivizumab-treated group and the vehicle control group. Next, presence or absence of matrix effects was determined for the therapeutic arm by comparing the vehicle control group with the control antibody group. Subsequently, efficacy of the CB017.5 treated group was assessed by comparison against the control group (vehicle and control antibody for the prophylactic and therapeutic arm, respectively) followed with a Holm-Bonferroni adjustment for in total five comparisons against the control group (an additional four unrelated treatment groups were included in this animal experiment to reduce the necessary number of animals and associated costs by sharing control groups, but they were not used to draw any conclusions in this study). The mock challenged vehicle group was added to give baseline values (lower limit of detection). Statistical analysis was performed using the Survival Package (Therneau, T. (2015) v2.38) for R (R Core Team (2016) v3.3.2).

Total histopathology scores were analyzed using the non-parametric Mann-Whitney test. First, model validity was determined by testing whether the difference between the mock infected and the RSV-infected vehicle treated group were significantly different (gatekeeper). Next, presence or absence of matrix effects were determined by comparing the vehicle group with the control mAb group. In the absence of matrix effects (i.e. no significant differences between vehicle and control mAb group), the palivizumab and CB017.5 groups were compared to vehicle with Sidak correction for multiple testing. With matrix effects, the palivizumab and CB017.5 groups were compared to the control mAb group.

Statistical analyses were performed using SAS version 9.2 (SAS Institute Inc., USA) and SPSS version 20 (SPSS Inc. USA). Statistical significance level was set at α = 0.05.

### Structure comparisons and analysis

Structural features were analyzed using the “Interfaces” feature of PDBePISA [65]. This analysis defined the antibody epitope and paratope, specific residues and molecular interactions involved in the interface, as well as the buried surface area. Structural comparison of the G peptides alone, as well as the antibody-bound structures, was accomplished by superimposing residues Cys173–Cys186 using the align feature in PyMOL [66].

## Supporting information

S1 Fig CB002.5 and CB017.5 epitope mapping determined by peptide binding. (A) Epitope maps of CB002.5 (blue) and CB017.5 (green) on the human RSV G sequence from strain A2, determined by peptide-binding data shown in (B) and (C). The single line indicates the mapped epitope, and the double line indicates residues which appear to be most important for binding. (B) The epitope of CB017.5 was determined by alanine-scanning mutagenesis. 32-residue peptides encompassing RSV G Phe165–Lys196 for subtype A (left) and subtype B (right) containing sequential alanine mutations were tested by ELISA for antibody binding. (C) Sequential short peptides encompassing the subtype B RSV G central conserved region were tested for antibody CB002.5 binding. The binding activity with each short peptide is shown as a vertical line proportional to the Pepscan ELISA signal. Each peptide grouping is presented in sequential order that shifts one amino acid at a time. The sequence of the highest-binding peptide per group, as well as the first peptide to demonstrate high binding in the 18-mer and 25-mer groups, are indicated with arrows. For panels (A–C), the paired cysteines that form the cystine noose are colored red (1–4) and green (2–3).
(TIF)

S2 Fig RSV neutralization in immortalized cell cultures in the absence of complement. Antibody-mediated neutralization data, based upon a firefly luciferase assay using RSV CL57, performed in A549 cells in the absence of complement when incubated with CB017.5 IgG (green) or CB002.5 IgG (blue). Palivizumab IgG (red) is included as a positive control.
(TIF)

S3 Fig Immunohistochemical staining of HBEC cultures. Microscopy images of HBEC cultures used in the neutralization assays. Cultures are immunohistochemically stained with alpha tubulin to indicate ciliated cells in red (A), Muc5AC to indicate mucin-containing goblet cells in green (B), or CD14 to indicate basal cells in blue (C).
(TIF)

S4 Fig CB002.5 and CB017.5 bind with decreased affinity to a reduced and alkylated form of the G peptide. SPR response curves of Fab CB002.5 (A) and Fab CB017.5 (B) binding to a reduced and alkylated form of the subtype A RSV G peptide. The raw data are plotted in black, and the calculated best fit to a 1:1 binding model is plotted in red. The equilibrium dissociation constant (*K*_D_) for each interaction is displayed above the respective SPR curve.
(TIF)
