# Supplementary figures and images for: Structural basis for recognition of the central conserved region of RSV G by neutralizing human antibodies
Source: PLoS Pathog. 2018 Mar 6;14(3):e1006935. doi: 10.1371/journal.ppat.1006935 (PMC5856423; doi:10.1371/journal.ppat.1006935)

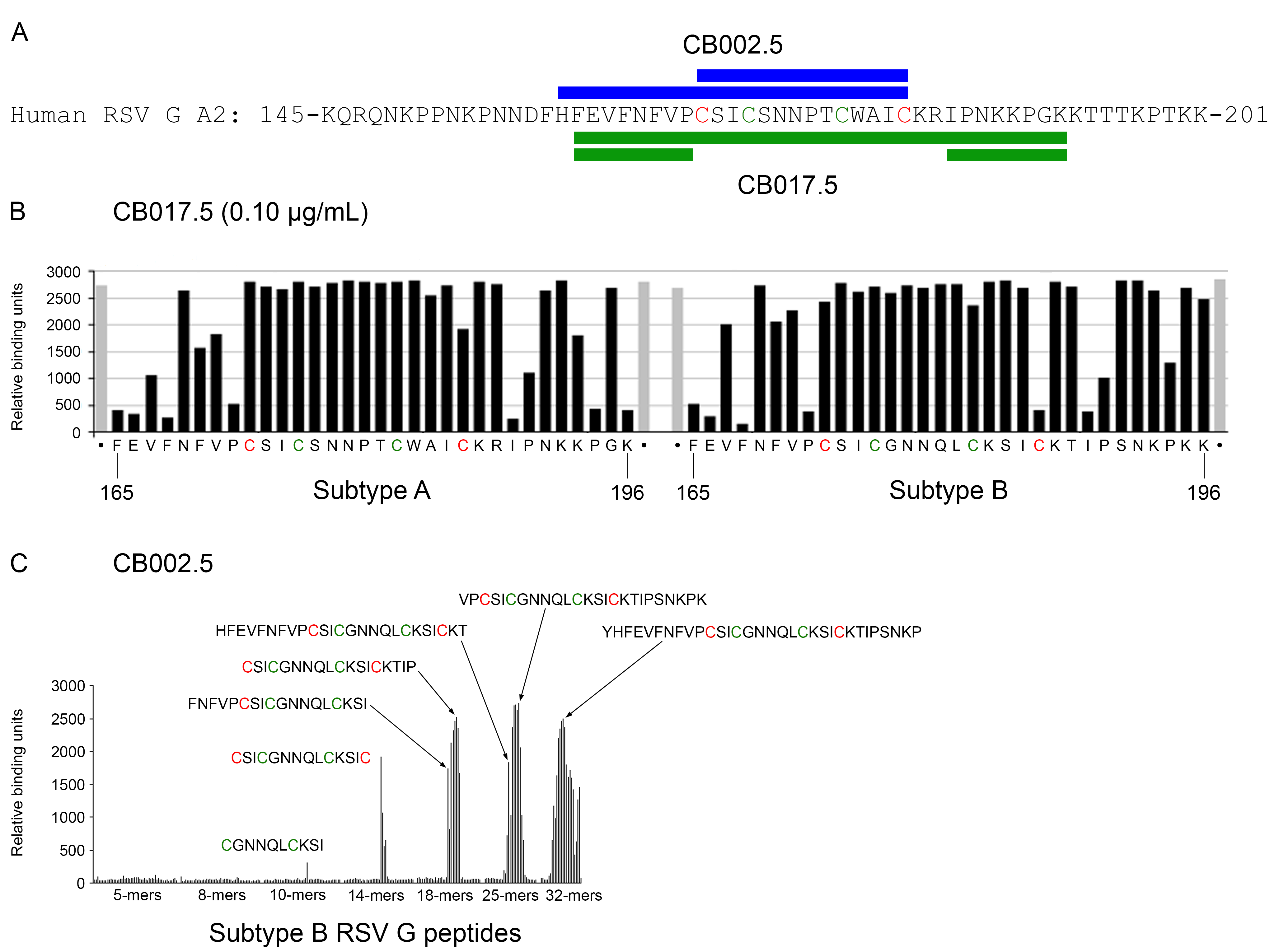

Supplement: S1 Fig — (A) Epitope maps of CB002.5 (blue) and CB017.5 (green) on the human RSV G sequence from strain A2, determined by peptide-binding data shown in (B) and (C). The single line indicates the mapped epitope, and the double line indicates residues which appear to be most important for binding. (B) The epitope of CB017.5 was determined by alanine-scanning mutagenesis. 32-residue peptides encompassing RSV G Phe165–Lys196 for subtype A (left) and subtype B (right) containing sequential alanine mutations were tested by ELISA for antibody binding. (C) Sequential short peptides encompassing the subtype B RSV G central conserved region were tested for antibody CB002.5 binding. The binding activity with each short peptide is shown as a vertical line proportional to the Pepscan ELISA signal. Each peptide grouping is presented in sequential order that shifts one amino acid at a time. The sequence of the highest-binding peptide per group, as well as the first peptide to demonstrate high binding in the 18-mer and 25-mer groups, are indicated with arrows. For panels (A–C), the paired cysteines that form the cystine noose are colored red (1–4) and green (2–3). (TIF) [file ppat.1006935.s001.tif]

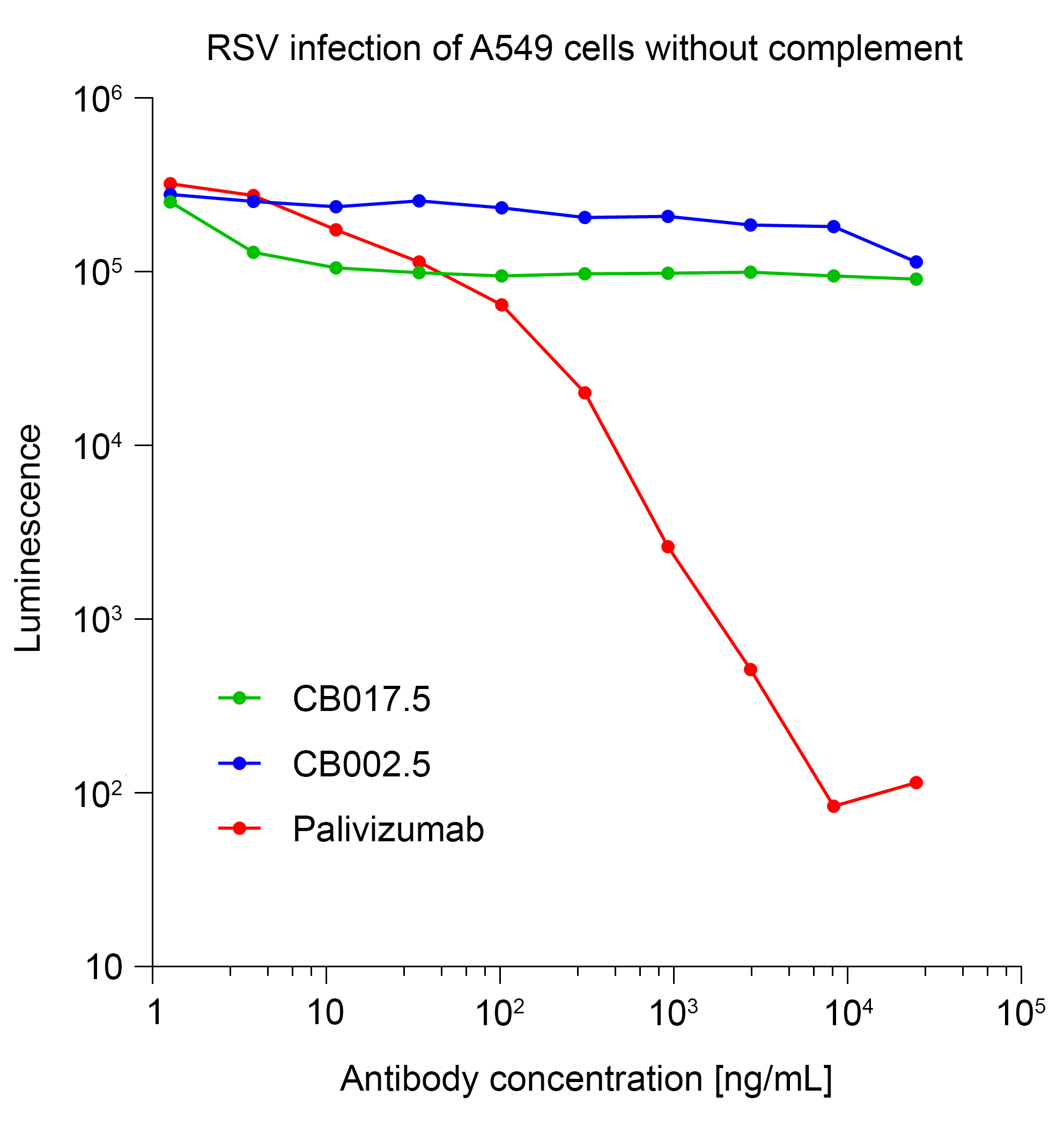

Supplement: S2 Fig — Antibody-mediated neutralization data, based upon a firefly luciferase assay using RSV CL57, performed in A549 cells in the absence of complement when incubated with CB017.5 IgG (green) or CB002.5 IgG (blue). Palivizumab IgG (red) is included as a positive control. (TIF) [file ppat.1006935.s002.tif]

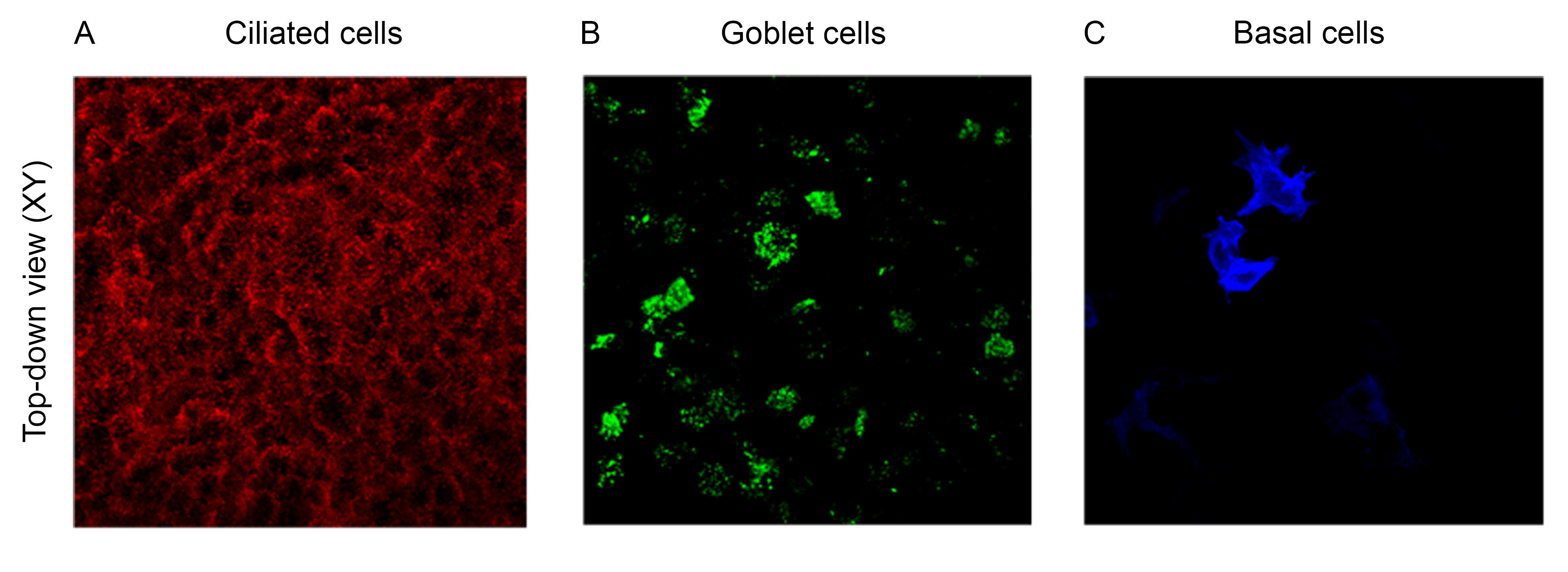

Supplement: S3 Fig — Microscopy images of HBEC cultures used in the neutralization assays. Cultures are immunohistochemically stained with alpha tubulin to indicate ciliated cells in red (A), Muc5AC to indicate mucin-containing goblet cells in green (B), or CD14 to indicate basal cells in blue (C). (TIF) [file ppat.1006935.s003.tif]

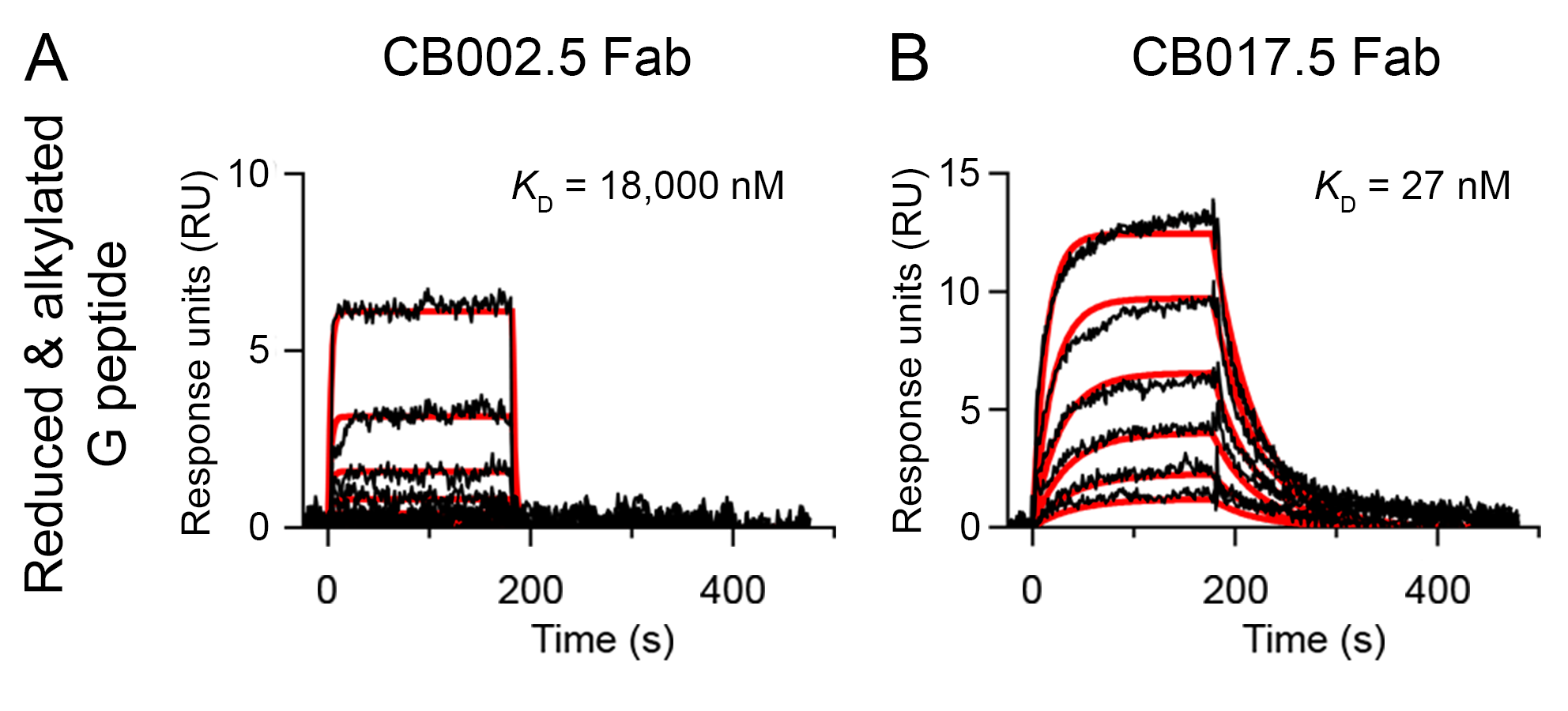

Supplement: S4 Fig — SPR response curves of Fab CB002.5 (A) and Fab CB017.5 (B) binding to a reduced and alkylated form of the subtype A RSV G peptide. The raw data are plotted in black, and the calculated best fit to a 1:1 binding model is plotted in red. The equilibrium dissociation constant (KD) for each interaction is displayed above the respective SPR curve. (TIF) [file ppat.1006935.s004.tif]
